# Genome-wide identification and analysis of the heat shock transcription factor family in moso bamboo (*Phyllostachys edulis*)

**DOI:** 10.1038/s41598-021-95899-3

**Published:** 2021-08-13

**Authors:** Bin Huang, Zhinuo Huang, Ruifang Ma, Jialu Chen, Zhijun Zhang, Kim Yrjälä

**Affiliations:** 1 State Key Laboratory of Subtropical Silviculture, Zhejiang A&F University, Zhejiang, China; 2 Zhejiang Provincial Collaborative Innovation Centre for Bamboo Resources and High Efficiency Utilization, Zhejiang A&F University, Zhejiang, China; 3 Department of Forest Sciences, University of Helsinki, Helsinki, Finland

**Keywords:** Genome, Genome informatics, Proteome informatics

## Abstract

Heat shock transcription factors (HSFs) are central elements in the regulatory network that controls plant heat stress response. They are involved in multiple transcriptional regulatory pathways and play important roles in heat stress signaling and responses to a variety of other stresses. We identified 41 members of the *HSF* gene family in moso bamboo, which were distributed non-uniformly across its 19 chromosomes. Phylogenetic analysis showed that the moso bamboo *HSF* genes could be divided into three major subfamilies; *HSFs* from the same subfamily shared relatively conserved gene structures and sequences and encoded similar amino acids. All *HSF* genes contained HSF signature domains. Subcellular localization prediction indicated that about 80% of the HSF proteins were located in the nucleus, consistent with the results of GO enrichment analysis. A large number of stress response–associated *cis*-regulatory elements were identified in the *HSF* upstream promoter sequences. Synteny analysis indicated that the *HSFs* in the moso bamboo genome had greater collinearity with those of rice and maize than with those of Arabidopsis and pepper. Numerous segmental duplicates were found in the moso bamboo *HSF* gene family. Transcriptome data indicated that the expression of a number of *PeHsf*s differed in response to exogenous gibberellin (GA) and naphthalene acetic acid (NAA). A number of *HSF* genes were highly expressed in the panicles and in young shoots, suggesting that they may have functions in reproductive growth and the early development of rapidly-growing shoots. This study provides fundamental information on members of the bamboo *HSF* gene family and lays a foundation for further study of their biological functions in the regulation of plant responses to adversity.

## Introduction

Plant growth is influenced by a variety of adverse environmental factors such as high temperature, salt stress, drought, and other abiotic stresses, as well as by biotic stresses such as diseases and pests. High temperature and drought are particularly important abiotic stresses that can promote chlorophyll degradation, damage chloroplast membrane mechanisms, and reduce photosynthetic efficiency^1^, thereby profoundly restricting plant growth, development, and function. Because of their sessile nature, plants cannot actively escape from stress and must rely on physiological and biochemical mechanisms to survive external stresses^2^. Therefore, they have evolved a series of complex and effective strategies to maintain normal physiology, metabolism, and growth under adverse conditions^3^. Transcription factor (TF) gene expression plays an important role in plant stress resistance^4^. For example, ABRE Binding Factor (ABF) and MYC TFs are directly involved in abscisic acid (ABA) and jasmonate (JA) signaling pathways^5^, and heat shock transcription factors (HSFs) are involved in the regulation of reactive oxygen species (ROS), calcium signaling, and other signal transduction pathways^6^. HSFs, a class of transcription factors widely found in eukaryotes, also have important roles in the receipt and transmission of signals, the recognition of heat shock elements (HSEs) and regulation of downstream genes, the stimulation of plant stress responses, and the promotion of heat tolerance^7^.

HSF transcription factors contain five basic functional domains: an N-terminal DNA-binding domain (DBD), an oligomerization domain (OD or HR-A/B), a nuclear localization signal (NLS), a nuclear export signal (NES), and in some cases, a C-terminal transcriptional activation domain (CTAD)^8^. The highly conserved DNA-binding domain (also known as the HSF domain) consists of three α-helix bundles and four strands of reverse-parallel β-folded layers that form a tight sphere. This structure ensures the specific binding of HSFs to HSEs in target gene promoters, thereby regulating the transcription and expression of heat shock genes^9^. The OD consists of two hydrophobic 7-peptide repeat regions, HR-A and HR-B, and HSFs are divided into three subfamilies (HsfA–C) based on differences in the number of amino acids inserted between HR-A and HR-B. HsfAs and HsfCs have 21 and 7 amino acid residues between HR-A and HR-B, respectively, whereas HsfBs have none^10,11^. The nuclear localization signal (NLS) is composed of basic amino acids and directs the transport of HSFs into the nucleus^12^, whereas the leucine-rich nuclear export signal (NES) facilitates their export from the nucleus to the cytoplasm^13^. The CTAD is the least conserved region and contains AHA motifs that consist of aromatic, large hydrophobic, and acidic amino acid residues. The AHA sequence is specific to the class A subfamily, and it is generally required for transcriptional activation. HsfBs and HsfCs do not contain AHA motifs and thus lack a transcriptional activation function^14^.

The first plant *HSF* gene was cloned from tomato in 1990. As more completed plant genome sequences became available, *HSF* family members were identified in Arabidopsis (21 *HSF* genes)^10^, tomato (26)^15^, cabbage (52)^16^, pepper (25)^17^, poplar (31)^18^, maize (25)^19^, and rice (25)^20^. Detailed functional studies of individual HSF subfamilies have been performed in Arabidopsis, tomato, and other model plants, and experiments have confirmed that HSFs have important roles in plant responses to heat and other abiotic stresses. Arabidopsis HsfAs were shown to be transcriptional activators, and AtHsfA1a regulates the synthesis of heat shock proteins (Hsps) during the heat-response phase to minimize plant growth damage at high temperatures^21^. The overexpression of Arabidopsis *AtHsfA2*^22^ and tomato *SlHsfA3*^23^ significantly increased both basal and acquired heat tolerance. The expression of *AtHsfA6a* increased nearly 150-fold under salt stress compared with control conditions^24^. By contrast, HsfB1 and HsfB2b from subfamily B were shown to be transcriptional repressors, and rice *OsHsfB2b* was found to significantly reduce plant salt tolerance after NaCl treatment^20,25^. Under drought stress, plants with upregulated expression of *AtHsfA1b* showed significant increases in yield and harvest index. Plants that overexpressed *HsfA1b* also had enhanced resistance to pathogenic bacteria, indicating that this gene is involved in the regulation of both abiotic and biotic stress resistance^26^.

Moso bamboo (*Phyllostachys edulis*) is a non-timber forestry species from the subfamily Bambusoideae of the family Poaceae and is native to China^27^. It has a wide distribution area and a high economic value, and it plays an important role in soil and water conservation, soil carbon sequestration and oxygen dynamics, and climate regulation^28^. However, water availability in moso bamboo forests is entirely dependent on natural precipitation, and its two most important water-demanding growth stages, ‘asparagus gestation’ in the autumn and ‘asparagus emergence’ in the spring, are most sensitive to heat and drought stress^28,29^. High temperatures and drought not only damage moso bamboo growth and development, thereby reducing the yield of bamboo shoots, but also reduce the carbon stocks of moso bamboo^30^. The intensification of global climate change is predicted to significantly alter the level and quality of ecological services to forests^31^, which will aggravate the effects of water stress on moso bamboo and significantly limit its yield.

To date, there have been no studies of the HSF transcription factor family in moso bamboo. Here, we used a bioinformatics approach to identify and characterize *HSF* genes and their encoded proteins in the moso bamboo genome^32,33^; we analyzed their evolutionary relationships, conserved domains, gene structures, promoter elements, duplication patterns, tissue-specific expression, and responses to external hormone treatments. Our results lay the groundwork for further study of heat stress response mechanisms in bamboo.

## Materials and methods

### Identification and sequence analysis of HSF proteins from moso bamboo

Genomic data from moso bamboo were downloaded from the *P. edulis* genome database (http://parrot.genomics.cn/gigadb/pub/10.5524/100001_101000/100,498/assembly_fasta/Bamboo.HIC.genome.fasta.gz). A hidden Markov model of the HSF DBD (PF00447) was obtained from the Pfam database (http://pfam.xfam.org/) and used as the seed model for an HMMER3 search (http://hmmer.janelia.org/) of the downloaded bamboo protein sequence data (*E* ≤ 10^−20^)^34^, and redundant genes were removed to produce a set of preliminary HSF candidate sequences. To verify that these candidates were HSFs, we used the normal mode of SMART (http://smart.embl-heidelberg.de/)^35^ sequence analysis with default search parameters and a Batch search (E < 0.001) of the Pfam (http://pfam.xfam.org)^36^ database to filter out sequences that lacked complete HSF_DNA-bind (PF00447.17) domains. The confirmed *HSF* genes were renamed according to their positions on the moso bamboo chromosomes.

Subcellular localization predictions were generated using CELLO with default parameters (http://cello.life.nctu.edu.tw/)^37^, and the ExPASy ProtParam tool (https://web.expasy.org/protparam/)^38^ was used with default parameters to predict protein physicochemical parameters such as molecular weight (MW) and isoelectric point (pI).

### Sequence alignment and phylogenetic tree construction

Whole genome information for Arabidopsis and rice was downloaded from the TAIR10 database (http://www.arabidopsis.org/index.jsp) and the Rice Genome Annotation Project database (http://rice.plantbiology.msu.edu). Maize and pepper genomic data were downloaded from the Ensembl database (http://asia.ensembl.org/index.html). Twenty-one Arabidopsis HSF proteins and 25 rice HSF proteins were identified from HMMER3 searches of the corresponding local protein databases^34^. The Arabidopsis and rice HSF sequences were combined with those from moso bamboo, a multiple protein sequence alignment was produced with ClustalX 2.0 (http://www.clustal.org/clustal2/). The comparison parameter is the multiple comparison mode (other parameters are default), and the resulting alignment was used to construct a maximum likelihood (ML) phylogenetic tree in MEGA 7.0 with 1000 bootstrap replicates^39^. Intraspecific classification of the moso bamboo HSF sequences was based on this interspecific phylogenetic tree.

The amino acid sequences of conserved domains were compared and edited using Jalview software (V2.10.5) (http://www.jalview.org/)^40^, and the Jalview output was submitted to JPred (http://www.compbio.dundee.ac.uk/jabaws) for protein secondary structure prediction using default parameters^41^.

### Gene structures, motif identification, and conserved domains

The intron–exon distributions of the moso bamboo *HSF* genes were obtained using GFF annotation files from the moso bamboo genome. Conserved amino acid sequences of HSF proteins were analyzed using the online MEME tool (http://meme-suite.org/)^42^. MEME analysis parameters included a minimum width ≥ 6, a maximum width of 50, and a motif number of 10; all other parameters were set to default values. Conserved domains in the HSFs were predicted using the NCBI Conserved Domain Database (https://www.ncbi.nlm.nih.gov/cdd/) (E-value < 0.001, other parameters set to defaults), and DOG 2.0 (http://dog.biocuckoo.org) was used for protein structure visualization with default parameters^43^.

### Chromosomal locations, genomic duplications, and Ka/Ks ratios

Chromosome lengths and gene locations were obtained from the moso genome annotation file, and MG2C v.2 (http://mg2c.iask.in/mg2c_v2.0/) was used to visualize the gene locations on chromosomes^44^. The moso bamboo protein sequences were aligned to one another or to the protein sequences from Arabidopsis, rice, maize, or pepper using TBtools software^45^. MCScanX^46^ was used with default parameters to identify gene duplication events and syntenic relationships among the HSF proteins, and the results were visualized using Circos and Dual Synteny Plot in TBtools^45^.

For Ka/Ks analysis, thirteen homologous gene pairs were identified by BLASTn using two criteria: (1) > 75% sequence similarity and (2) an alignable region > 75% of the length of the longer sequence^47^. KaKs_Calculator2.0 was used to calculate the synonymous substitution rate (Ks), nonsynonymous substitution rate (Ka), and Ka/Ks ratio between homologous gene pairs^48^. Evolutionary divergence times within the bamboo *HSF* gene family were calculated using the bamboo-specific divergence time formula T = Ks/2λ (where λ = 6.5 × 10^−9^)^32^.

### Identification of *cis*-acting elements

PlantCARE (http://bioinformatics.psb.ugent.be/webtools/plantcare/html/) was used to identify *cis*-acting elements in the 1,500-bp promoter region upstream of each gene’s transcription start site, and the results were visualized using TBtools^49^.

### Expression analysis of the HSF genes

Replicated transcriptomic data from different plant organs (roots, rhizomes, panicles, and leaves), bamboo shoots at different germination stages (20, 50, and 100 cm), and seedling root tissues treated with 5 μM gibberellic acid (GA) or 5 μM naphthalene acetic acid (NAA) were obtained from the NCBI Sequence Read Archive (https://www.ncbi.nlm.nih.gov/sra) and the European Nucleotide Archive (https://www.ebi.ac.uk/ena/browser/home). In total, 31 transcriptomic datasets were downloaded (accession numbers SRP119924, SRP012682, SRP109631, ERR105067, ERR105068, ERR105069, ERR105070, ERR105071, ERR105072, ERR105073, ERR105074, ERR105075, and ERR105076.) Transcriptome data in the form of transcripts per million reads (TPM) were log_10_-transformed and imported into TBtools, where Amazing Heatmap was used to generate expression heatmaps.

### Three-dimensional (3D) structural modeling of HSF family proteins

The PDB database (http://www.rcsb.org/) was used to retrieve protein models homologous to those of the HSF proteins. Swiss Model (https://www.swissmodel.expasy.org/) was then used with default parameters to predict the protein tertiary structures by homology modeling, and the quality of the resulting models was assessed using SAVES v.5.0 (http://servicesn.mbi.ucla.edu/SAVES/).

### Protein interaction network predictions and GO enrichment analyses

The HSF protein sequences were uploaded to the STRING database (https://string-db.org/) for node comparison, and relationships among important proteins were predicted based on rice protein interactions. Cytoscape (V3.7.1) was used to visualize the resulting network^50^.

GOATOOLS (http://github.com/tanghaibao/GOatools)^51^ was used to assign GO annotations to HSFs, and Fisher's exact test was used to identify biological functions enriched in the PeHsfs relative to the full GO database. A Bonferroni^52^ multiple testing correction was used to minimize false positives, and functions were considered to be significantly enriched when their Bonferroni-corrected *P*-values (Padjust) were < 0.05.

## Results

### Identification of HSF genes in moso bamboo

Forty-four putative *HSF* candidate genes were obtained from an HMMER3 search of the bamboo protein database using the plant HSF-type DBD model (Pfam PF00447) with an *E*-value threshold of ≤ 10^−20^. We removed redundant genes and verified the presence of conserved domains and motifs to arrive at a final set of 41 *HSF* family members. The genes were renamed *PeHsf01*–*PeHsf41* based on their positions on the chromosomal scaffolds (Table 1).

**Table 1:** Detailed information on 41 *PeHsf* genes and their encoded proteins.

Gene name	Gene ID	Chromosome location	Size (aa)	MW (kDa)	pI	Stability	A.I	GRAVY	Predicted location
*PeHsf01*	PH02Gene27951.t1	S3:17095591–17101377	292	31.90	9.21	U	66.88	− 0.601	Nuclear
*PeHsf02*	PH02Gene22062.t1	S3:17300229–17302543	385	41.30	8.86	U	68.83	− 0.359	Nuclear
*PeHsf03*	PH02Gene32485.t1	S4:5279994–5282107	159	17.49	5.82	U	68.62	− 0.484	Nuclear
*PeHsf04*	PH02Gene23029.t2	S5:34339214–34343740	382	43.06	4.72	U	83.25	− 0.539	Cytoplasmic
*PeHsf05*	PH02Gene21935.t1	S6:50181002–50183068	276	29.02	6.98	S	63.73	− 0.264	Extracellular
*PeHsf06*	PH02Gene24187.t1	S7:43016455–43019591	445	49.96	4.95	U	67.46	− 0.626	Nuclear
*PeHsf07*	PH02Gene17129.t1	S8:53174946–53177158	348	37.56	4.87	U	81.49	− 0.250	Cytoplasmic
*PeHsf08*	PH02Gene21435.t1	S9:57449663–57452744	453	51.38	5.23	U	66.29	− 0.649	Nuclear
*PeHsf09*	PH02Gene35749.t1	S10:3519871–3522549	300	33.58	6.35	U	66.33	− 0.653	Nuclear
*PeHsf10*	PH02Gene31077.t2	S13:91174631–91176831	383	40.63	5.40	U	69.16	− 0.380	Nuclear
*PeHsf11*	PH02Gene16951.t1	S13:117714221–117720267	362	41.14	4.98	U	78.37	− 0.698	Nuclear
*PeHsf12*	PH02Gene22176.t1	S14:33627815–33630503	319	35.90	5.76	U	74.92	− 0.490	Cytoplasmic
*PeHsf13*	PH02Gene40343.t1	S14:40539424–40541046	311	34.31	5.73	U	71.25	− 0.388	Nuclear
*PeHsf14*	PH02Gene18810.t1	S14:54857540–54858808	249	27.71	9.19	U	69.92	− 0.310	Cytoplasmic
*PeHsf15*	PH02Gene15090.t1	S14:57115384–57117986	443	49.15	5.24	U	64.94	− 0.732	Nuclear
*PeHsf16*	PH02Gene17948.t1	S15:38771524–38774540	279	31.40	5.69	U	66.74	− 0.638	Nuclear
*PeHsf17*	PH02Gene50090.t1	S15:61679276–61684197	409	45.25	4.84	U	72.47	− 0.631	Nuclear
*PeHsf18*	PH02Gene03550.t1	S15:72232636–72234806	315	36.53	5.05	U	73.4	− 0.696	Nuclear
*PeHsf19*	PH02Gene11863.t1	S15:94500097–94503858	342	38.49	5.32	U	63.54	− 0.798	Nuclear
*PeHsf20*	PH02Gene14728.t1	S16:34492266–34494798	444	49.50	5.34	U	59.35	− 0.856	Nuclear
*PeHsf21*	PH02Gene30851.t1	S16:37537769–37539234	247	26.77	8.93	S	72.43	− 0.264	Mitochondrial
*PeHsf22*	PH02Gene20958.t1	S16:54912146–54913593	310	34.09	5.86	U	74.29	− 0.343	Nuclear
*PeHsf23*	PH02Gene48407.t1	S16:66912172–66914576	319	35.61	5.67	U	71.88	− 0.455	Cytoplasmic
*PeHsf24*	PH02Gene29649.t1	S17:49074756–49079687	494	54.90	4.99	U	79.17	− 0.573	Nuclear
*PeHsf25*	PH02Gene33162.t1	S17:54687050–54691243	185	53.80	5.01	U	58.8	− 0.749	Nuclear
*PeHsf26*	PH02Gene32696.t1	S17:80486587–80489026	288	30.99	5.81	U	63.33	− 0.456	Chloroplast
*PeHsf27*	PH02Gene26216.t1	S18:26517557–26519682	360	38.81	5.04	U	65.69	− 0.649	Nuclear
*PeHsf28*	PH02Gene01234.t1	S18:37635773–37642186	296	32.43	8.85	U	64.63	− 0.605	Nuclear
*PeHsf29*	PH02Gene21649.t1	S18:38024325–38025852	383	40.93	8.65	U	64.63	− 0.381	Nuclear
*PeHsf30*	PH02Gene30467.t1	S20:5525280–5530407	485	53.62	5.19	U	57.01	− 0.764	Nuclear
*PeHsf31*	PH02Gene40145.t1	S20:31531508–31534036	334	36.32	6.67	U	67.99	− 0.441	Chloroplast
*PeHsf32*	PH02Gene05710.t1	S20:52393478–52397181	512	56.33	5.06	U	77.95	− 0.501	Nuclear
*PeHsf33*	PH02Gene08915.t1	S21:1225128–1229768	507	55.29	4.88	U	73.16	− 0.448	Nuclear
*PeHsf34*	PH02Gene06729.t1	S21:9161083–9164543	358	40.28	5.40	U	66.15	− 0.812	Nuclear
*PeHsf35*	PH02Gene14439.t1	S21:64405316–64407893	278	31.78	5.70	U	66.22	− 0.695	Nuclear
*PeHsf36*	PH02Gene26611.t1	S21:96626845–96633311	392	43.64	5.14	U	72.37	− 0.598	Nuclear
*PeHsf37*	PH02Gene09168.t2	S21:105791731–105794734	362	41.44	5.05	U	72.49	− 0.692	Nuclear
*PeHsf38*	PH02Gene24117.t1	S22:2617776–2619602	372	39.87	4.91	U	71.75	− 0.412	Nuclear
*PeHsf39*	PH02Gene46516.t1	S22:15546435–15548028	316	39.36	9.31	U	73.32	− 0.345	Nuclear
*PeHsf40*	PH02Gene01898.t1	S23:17548422–17549945	292	31.29	5.35	U	59.59	− 0.771	Nuclear
*PeHsf41*	PH02Gene00565.t1	S24:57049346–57050791	321	34.22	5.22	U	59.13	− 0.743	Nuclear

*MW* molecular weight, *pI* isoelectric point, *A.I.* aliphatic index, *GRAVY* grand average of hydropathicity score.

The predicted physicochemical properties of the amino acid sequences showed that the 41 HSF genes encoded proteins containing 159 (*PeHsf03*) to 512 (*PeHsf30*) amino acids and their molecular weights ranged from 17.49 (*PeHsf03*) to 56.33 kDa (*PeHsf32*). Approximately 90% of the HSF proteins had molecular weights of 30–50 kDa. Their predicted isoelectric points (pI) ranged from 4.72 (*PeHsf04*) to 9.31 (*PeHsf39*). Instability index calculations predicted that 39 (95%) of the HSF proteins were stable in vitro, with the exceptions of *PeHsf05* and *PeHsf21*, which had instability indices of 32.96 and 37.27, respectively. Aliphatic amino acid indices showed that the thermal stability of the proteins ranged from 57.01 to 83.25, indicating that differences in their thermal stability were relatively minor. The grand average of hydropathicity (GRAVY) scores of all HSF proteins were negative, indicating that they were hydrophilic proteins. CELLO subcellular localization predictions suggested that about 80% of the HSF proteins were located in the nucleus.

### Phylogenetic analysis and sequence alignment of the *PeHsf* proteins

**Figure 1:**
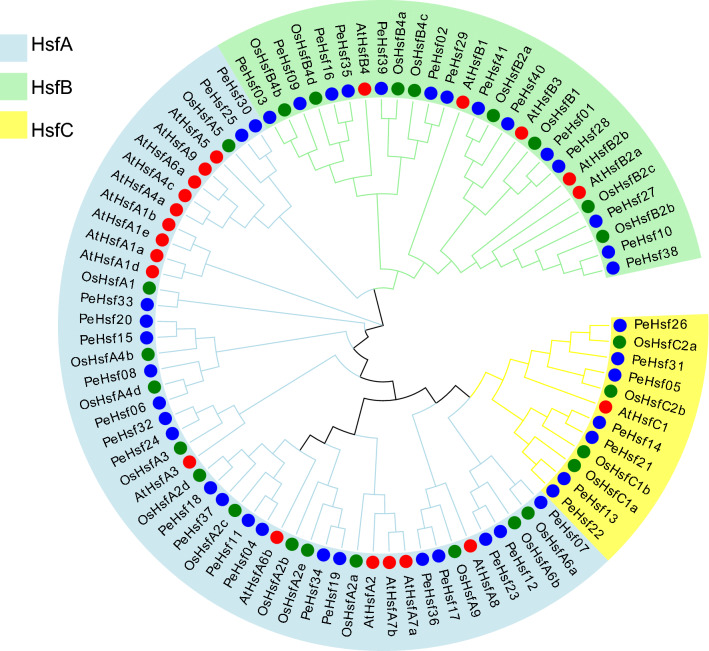
Phylogenetic analysis of full-length HSF protein sequences from *Phyllostachys edulis* (Pe, bamboo), *Arabidopsis thaliana* (At, Arabidopsis), and *Oryza sativa* (Os, rice). ClustalX was used to build a multiple sequence alignment, and MEGA7.0 (https://www.megasoftware.net/) was used to construct a maximum likelihood (ML) phylogenetic tree with 1000 bootstrap replicates. Blue, red, and green circles indicate bamboo, Arabidopsis, and rice sequences, respectively. Evolview (https://evolgenius.info//evolview-v2/#login) online URL for beautification.

To clarify the evolutionary relationships among the HSF proteins, amino acid sequences of 41 moso bamboo HSFs, 25 rice HSFs, and 21 Arabidopsis HSFs were used to construct an ML phylogenetic tree^53^ (Fig. 1 and Supplementary Table S1). Based on well-established Arabidopsis and rice HSF family classifications^54^, the HSF proteins were divided into three major subfamilies, HsfA (blue), HsfB (green), and HsfC (yellow). The HsfA subfamily was the largest, with 48 members across the three species, whereas the HsfC subfamily was the smallest, with 12 members. There was only one Arabidopsis HsfC family member (AtHsfC1), suggesting that the C subfamily had expanded in monocots. The interspecific phylogenetic tree indicated that the PeHsfs included members of all three subfamilies: 20 HsfAs, 14 HsfBs, and 7 HsfCs.

**Figure 2:**
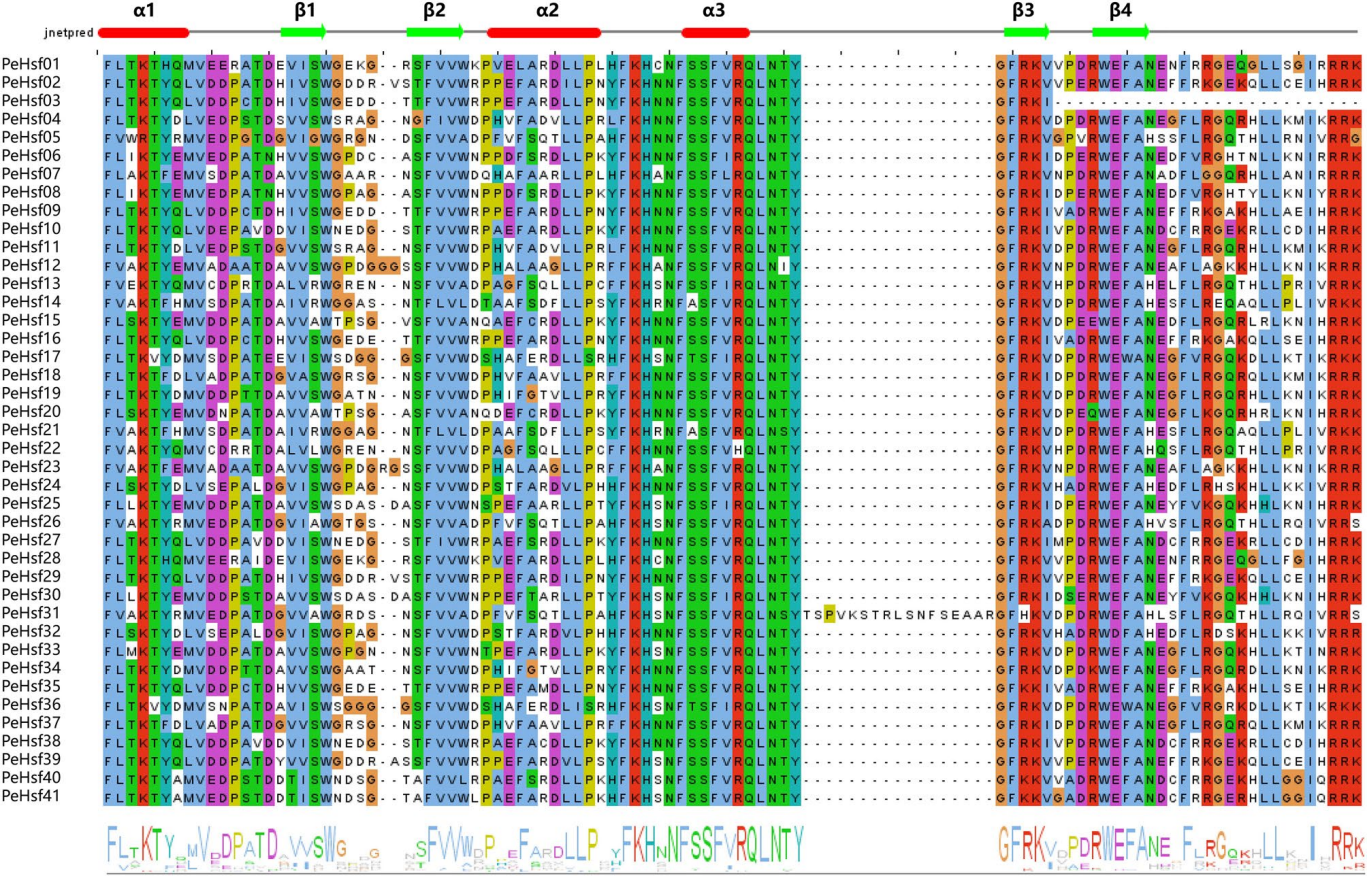
Multiple sequence alignment of the DNA-binding domains (DBDs) of HSF proteins from moso bamboo. Secondary structure elements of the DBDs (α1-β1-β2-α2-α3-β3-β4) based on JNet structure predictions are shown above the alignment (see Methods). α-helices are indicated by red bars, and β-folds are indicated by green arrows. The amino acid sequences of conserved domains were compared and edited using Jalview software (V2.10.5) (http://www.jalview.org/)^2^, and the Jalview output was submitted to JPred (http://www.compbio.dundee.ac.uk/jabaws) for protein secondary structure prediction using default parameters(Supplementary Table S2) .

A multiple sequence alignment of the 41 PeHsf proteins was generated to investigate the presence and locations of conserved protein domains. All HSF family members contained a highly conserved DBD at the amino terminus that consisted of approximately 100 amino acids (Fig. 2). The protein secondary structure was predicted to contain three α-helix bundles (α1–α3) and four reverse-parallel β-folds (β1–β4). The DBD domain can specifically recognize and precisely localize heat stress elements, and similar phenomena are found in other plants.

### Gene structures, motif identification, and conserved domain analysis

**Figure 3:**
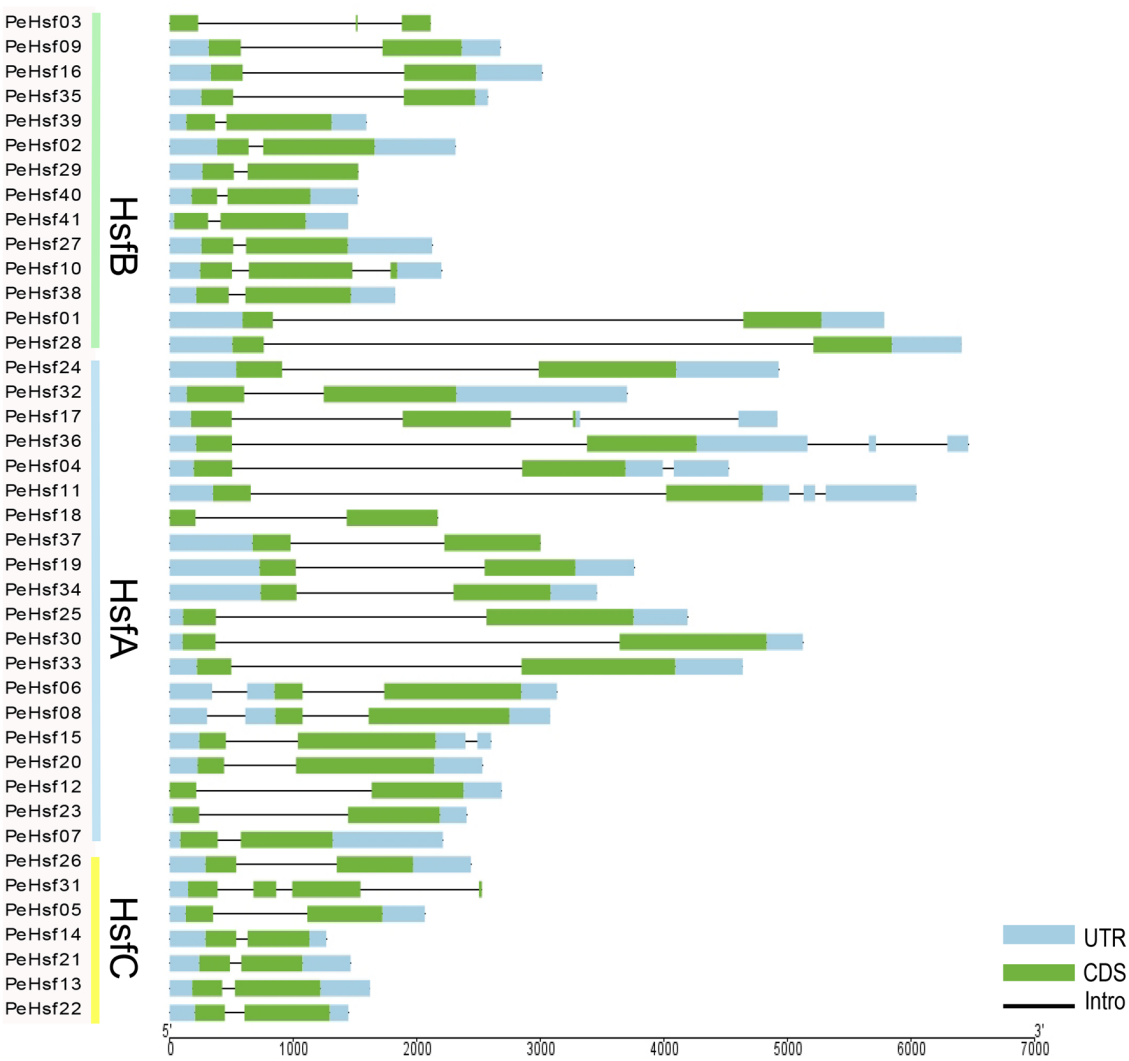
Intron–exon structure of the *HSF* genes in moso bamboo. Green boxes represent exons (CDS), black lines represent introns, and light blue boxes represent 5′ and 3′ untranslated regions. Visualized by TBtools (v1.0697): Visualize Gene Structure (https://github.com/CJ-Chen/TBtools/releases).

The number of introns in each moso bamboo *HSF* gene ranged from one to three (Fig. 3). Approximately 75% of the genes contained a single intron, whereas six genes contained two introns and four contained three introns. With the exception of *PeHsf31*, all *HsfCs* contained only one intron. Structures of *HSF* genes from the same subfamily were generally similar.

**Figure 4:**
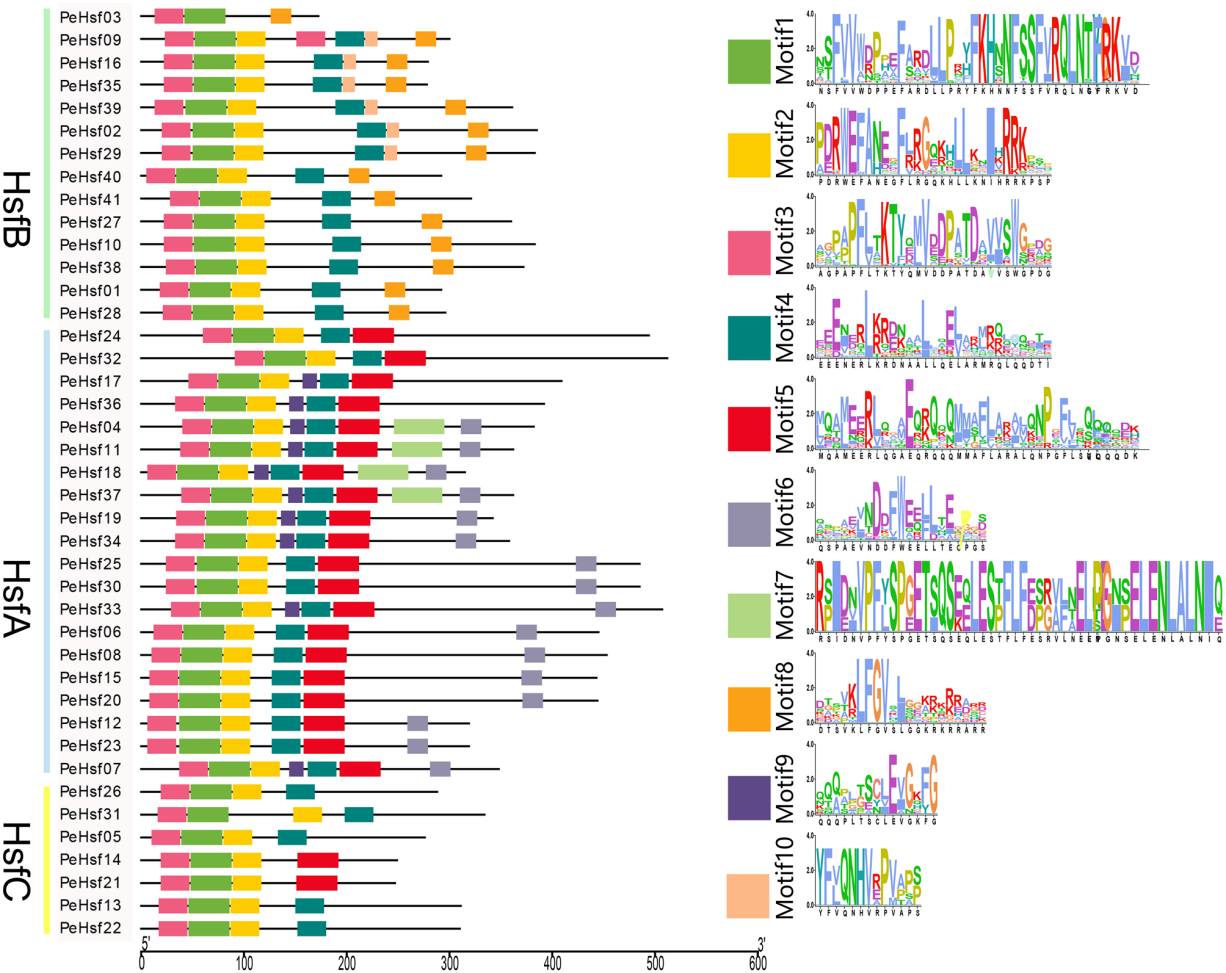
Distribution of conserved motifs of HSF proteins from moso bamboo. The scale bar at the bottom indicates the protein lengths, and sequence logos for each conserved motif are shown at right. TBtools (v1.0697) (https://github.com/CJ-Chen/TBtools/releases): Visualize MEME/MAST Motif pattern for visualization. The XML file obtained by the MEME online tool is obtained by visualizing the Batch MEME Motifs Viz plugin in TBtools (v1.0697).

We identified up to 10 highly conserved motifs in each HSF protein using MEME (Fig. 4). All HSFs contained motifs 1–3, which constituted the most highly conserved portions of the DBD. The relative positions of the motifs were similar for most sequences, with the exception of the HsfB *PeHsf03*, which lacked motif 2. In addition, both class B and C subfamily members did not contain motif 6, which is associated with transcriptional activation.

**Figure 5﻿:**
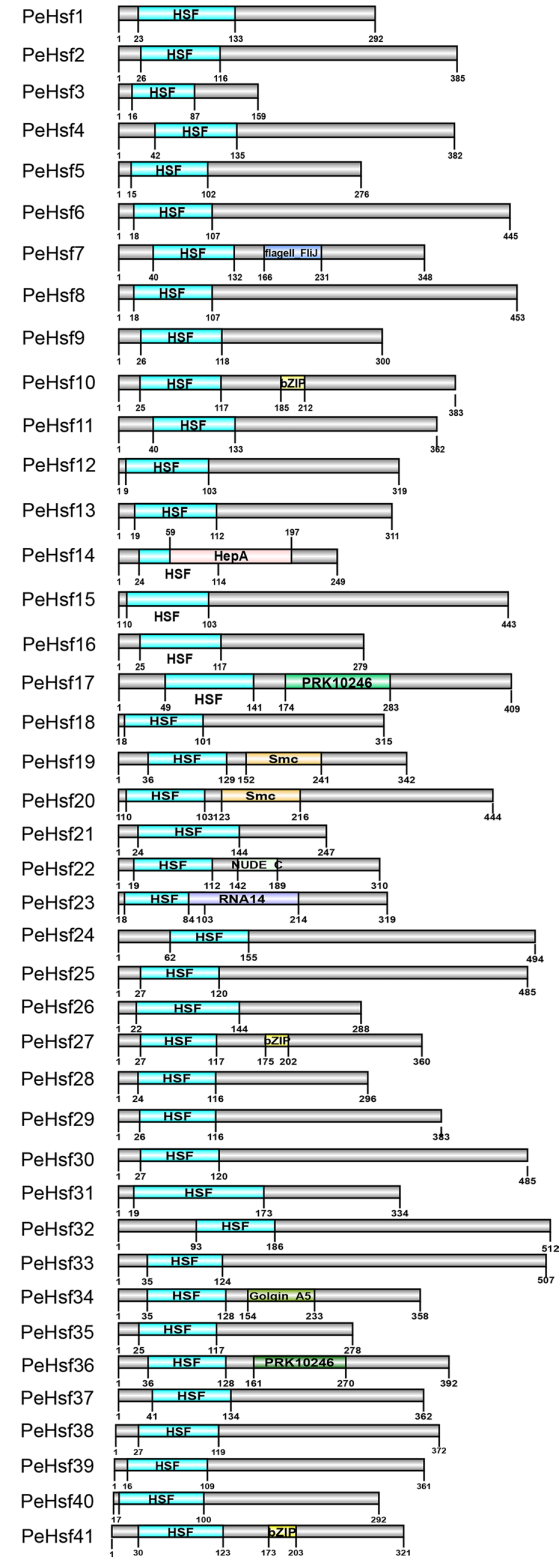
Conserved domain predictions for 41 PeHsf proteins. The gray bars represent the length of each protein sequence, and conserved domains are represented by colored boxes. DOG 2.0 (http://dog.biocuckoo.org) was used for protein structure visualization with default parameters.

The structures of the PeHsf proteins were further characterized using the NCBI Conserved Domain Database (Fig. 5). All PeHsf proteins contained highly conserved DBDs at the N terminus between amino acids 7 and 155, the presence of which served as confirmation of their identity as HSF proteins. Some proteins contained additional conserved domains. For example, *PeHsf10*, *PeHsf27*, and *PeHsf41* contained a bZIP-like transcription factor domain, and *PeHsf17* and *PeHsf36* contained a PRK10246 domain. Therefore, multiple functions may have evolved in some members of the moso bamboo HSF family.

### Chromosomal distribution and synteny analysis of the *PeHsf* genes

**Figure 6:**
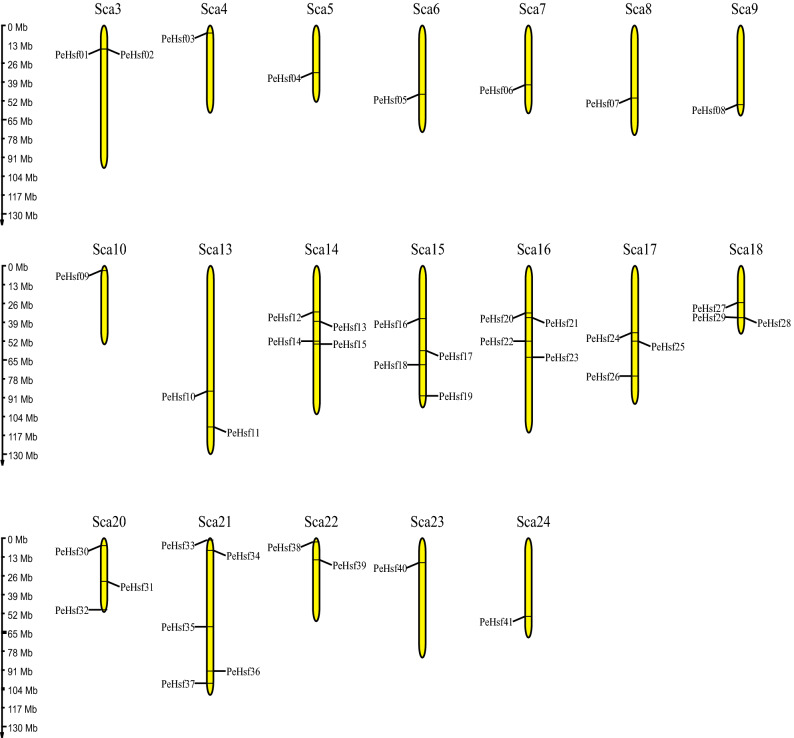
The distribution of *PeHsf* genes on the moso bamboo chromosome scaffolds. MG2C v.2 (http://mg2c.iask.in/mg2c_v2.0/) was used to visualize the gene locations on chromosomes.

The *PeHsf* genes were non-uniformly distributed across the 19 chromosome scaffolds of moso bamboo (Fig. 6). The largest number were found on scaffold 21 (5), followed by scaffolds 14, 15, and 16 (4), scaffolds 17, 18, and 20 (3), and scaffolds 3, 13, and 22 (2). All other chromosomes contained a single *PeHsf* gene. Small gene clusters were found on scaffolds 3 and 18, based on the definition of gene clusters^55^.

**Figure 7:**
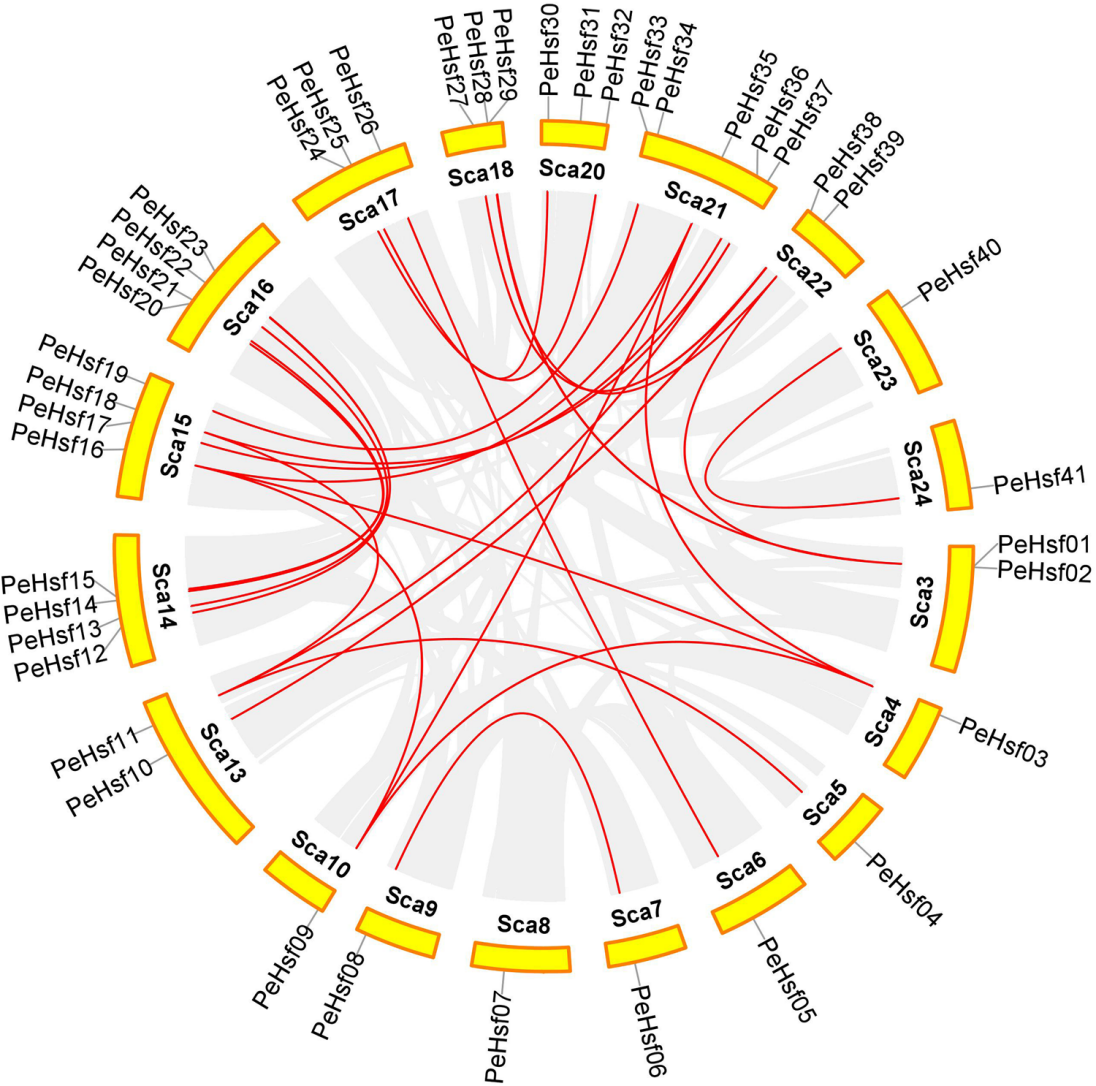
Chromosomal distribution and interchromosomal relationships of *PeHsf* genes. Gray lines indicate syntenic blocks within the moso bamboo genome, and red lines represent duplicate *PeHsf* gene pairs. Visualization using Circos in TBtools (v1.0697) (https://github.com/CJ-Chen/TBtools/releases).

Gene duplication events are prevalent in all species; they can give rise to new functional genes and drive species evolution^56^. We therefore used MCScanX genome synteny analysis to explore duplications within the moso bamboo *HSF* gene family (Fig. 7). Twenty-seven gene pairs appeared to have arisen from segmental duplications, and only *PeHsf07* and *PeHsf31* had no duplicates in the genome. Previous studies have shown that two or more highly similar genes located in close proximity to one another are likely to be tandem duplicates^57^. As shown in Fig. 6, two such pairs, *PeHsf01*–*PeHsf02* and *PeHsf28*–*PeHsf29*, were present on scaffolds 3 and 18, respectively.

**Figure 8:**
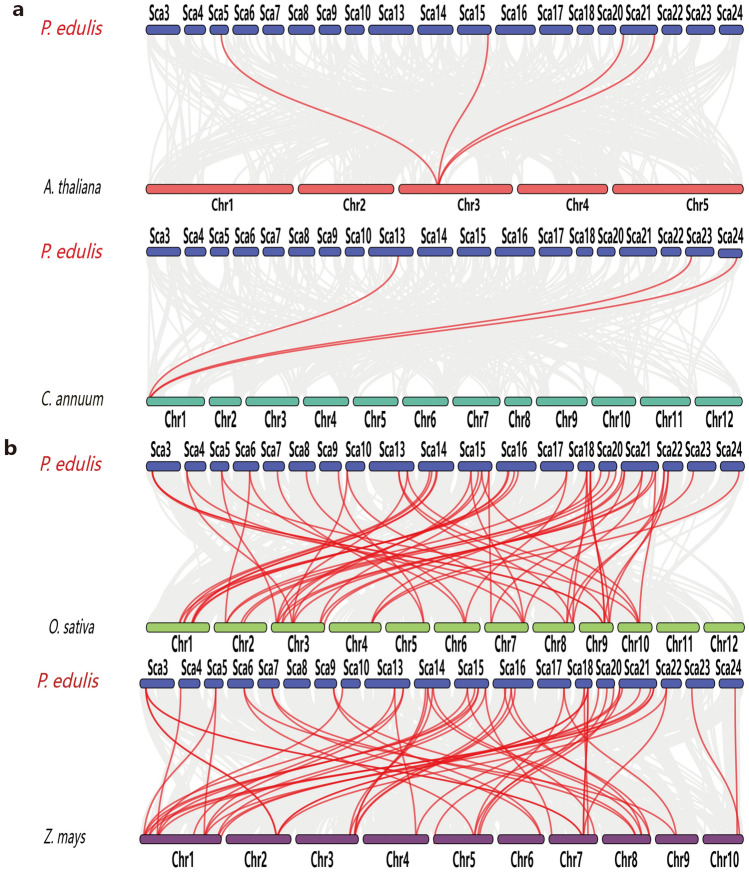
Synteny analysis of the moso bamboo genome with two monocot (**a**) and two dicot (**b**) plant genomes. The gray lines represent aligned blocks between the paired genomes, and the red lines indicate syntenic *HSF* gene pairs. Visualization was performed using Dual Synteny Plot in TBtools (v1.0697).

To further investigate gene duplications in the *HSF* gene family, we performed genome-to-genome synteny analysis between moso bamboo and four representative plants (Supplementary Table S3). These included two dicots, Arabidopsis and *Capsicum annuum* (pepper) (Fig. 8a), and two monocots, *O. sativa* (rice) and *Zea mays* (maize) (Fig. 8b). Four, 3, 58, and 52 moso bamboo *HSF* genes were syntenic with those of Arabidopsis, pepper, rice, and maize, respectively. Furthermore, the rice *HSF* genes all had corresponding orthologs in moso bamboo, and most of them had more than two orthologs, suggesting that moso bamboo has undergone additional whole-genome duplication events during its evolution.

To investigate evolutionary constraints and selection pressures on the *PeHsf* genes, we calculated Ka, Ks, and Ka/Ks for 13 homologous *PeHsf* gene pairs (Table 2). The synonymous substitution rate (Ks) represents the background base substitution rate, and Ks values can therefore be used to predict the timing of whole-genome duplication events. The Ks values of the *PeHsf* gene pairs ranged from 0.1023 to 1.0833, indicating that a large-scale *PeHsf* gene duplication event occurred as early as 83.33 million years ago (MYA) and as recently as 7.86 MYA. The Ka/Ks values of the gene pairs were all less than 1.0, and these genes may have undergone strong purifying selection during evolution.

**Table 2:** The Ka/Ks values of homologous *PeHsf* gene pairs.

Gene pair	Ka	Ks	Ka/Ks	MYA
*PeHsf01*–*PeHsf28*	0.0315	0.3727	0.0845	28.6692
*PeHsf04*–*PeHsf11*	0.9711	1.0833	0.8964	83.3330
*PeHsf06*–*PeHsf08*	0.4283	0.6143	0.6972	47.2538
*PeHsf15*–*PeHsf20*	0.0550	0.1113	0.4936	8.5615
*PeHsf14*–*PeHsf21*	0.0342	0.3268	0.1048	25.1384
*PeHsf12*–*PeHsf23*	0.0582	0.1023	0.5690	7.8692
*PeHsf18*–*PeHsf37*	0.9907	1.0268	0.9649	78.9846
*PeHsf19*–*PeHsf34*	0.0430	0.1212	0.3546	9.3230
*PeHsf16*–*PeHsf35*	0.0350	0.2788	0.1254	21.4461
*PeHsf17*–*PeHsf36*	0.0557	0.1062	0.5248	8.1692
*PeHsf22*–*PeHsf13*	0.0577	0.2749	0.2100	21.1461
*PeHsf24*–*PeHsf32*	0.9893	1.0366	0.9544	79.7384
*PeHsf25*–*PeHsf30*	0.0420	0.1145	0.3670	8.8076

*Ka* non-synonymous substitution rate, *Ks* synonymous substitution rate, *MYA* million years ago.

### Promoter *cis*-element analysis of *PeHsf* genes

**Figure 9:**
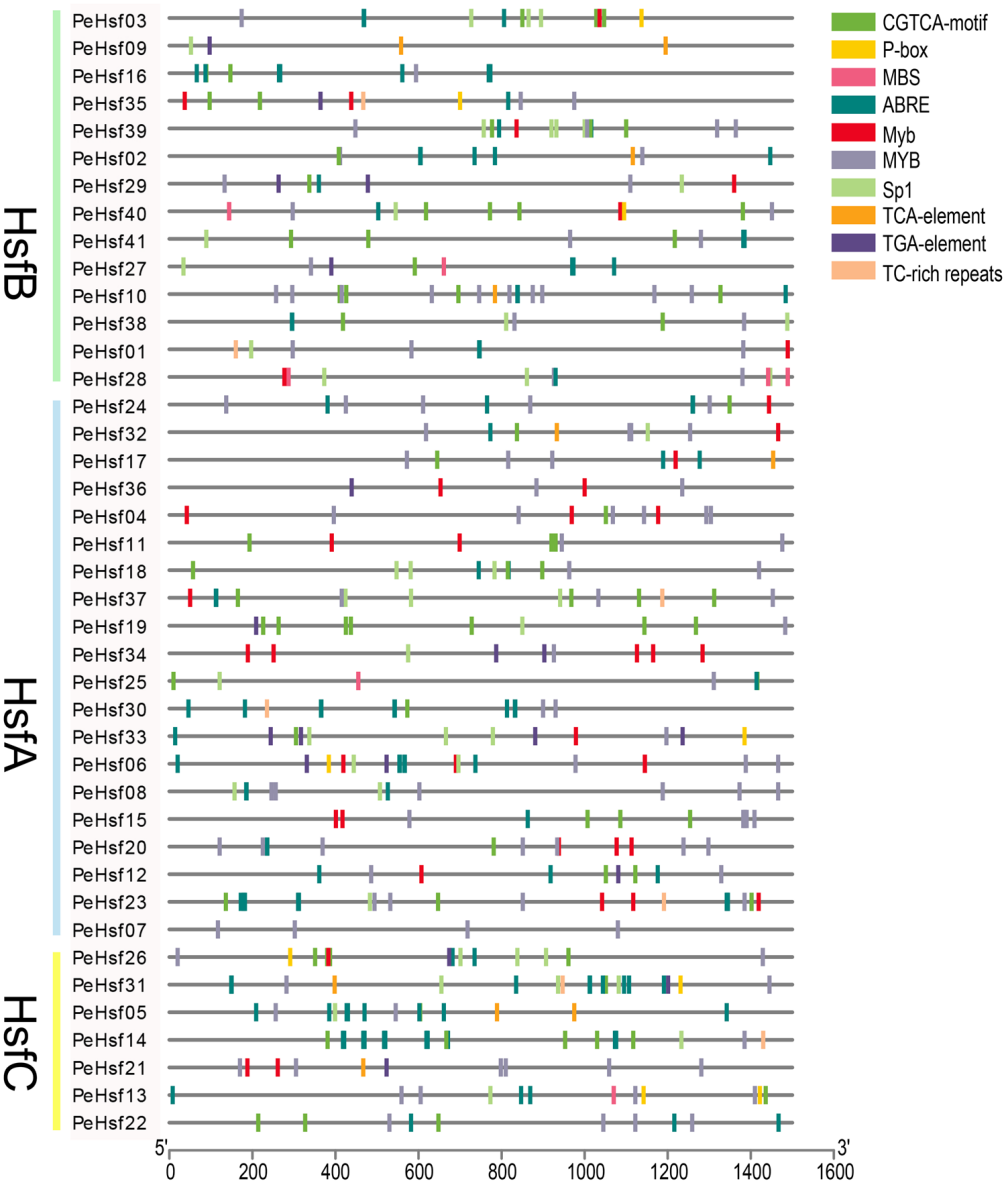
Diagram of *cis*-acting elements identified in the 1,500-bp upstream promoter regions of the *PeHsf* genes. Rectangular boxes of different colors represent individual *cis*-acting elements, and some elements overlap with one another. PlantCARE (http://bioinformatics.psb.ugent.be/webtools/plantcare/html/) was used to identify cis-acting elements in the 1500-bp promoter region upstream of each gene’s transcription start site, and the results were visualized using TBtools (v1.0697).

*cis*-acting elements are non-coding DNA sequences in gene promoters that regulate the transcription of their associated genes. We identified ten *cis*-acting elements in the 1500 base pairs upstream of the *PeHsf* genes using PlantCARE software (Fig. 9). Most were stress-response elements, and all promoters except that of *PeHsf09* contained at least one MYB drought stress response element. The next most common element was the ABRE element, which is involved in abscisic acid responses. Other elements present in the *PeHsf* promoters included the MBS element (involved in drought, high salt, and low temperature responses), the TC-rich repeat (involved in defense and stress responses), the SP1 light response element, the P-box and TGA gibberellin response elements, the TCA salicylic acid response element, and the CGTCA motif (involved in methyl jasmonate response). These results suggest that the expression of moso bamboo *HSF* genes is regulated by *cis*-elements associated with plant developmental processes and abiotic stress responses.

### Transcription profiles of the *PeHsf* genes

**Figure 10:**
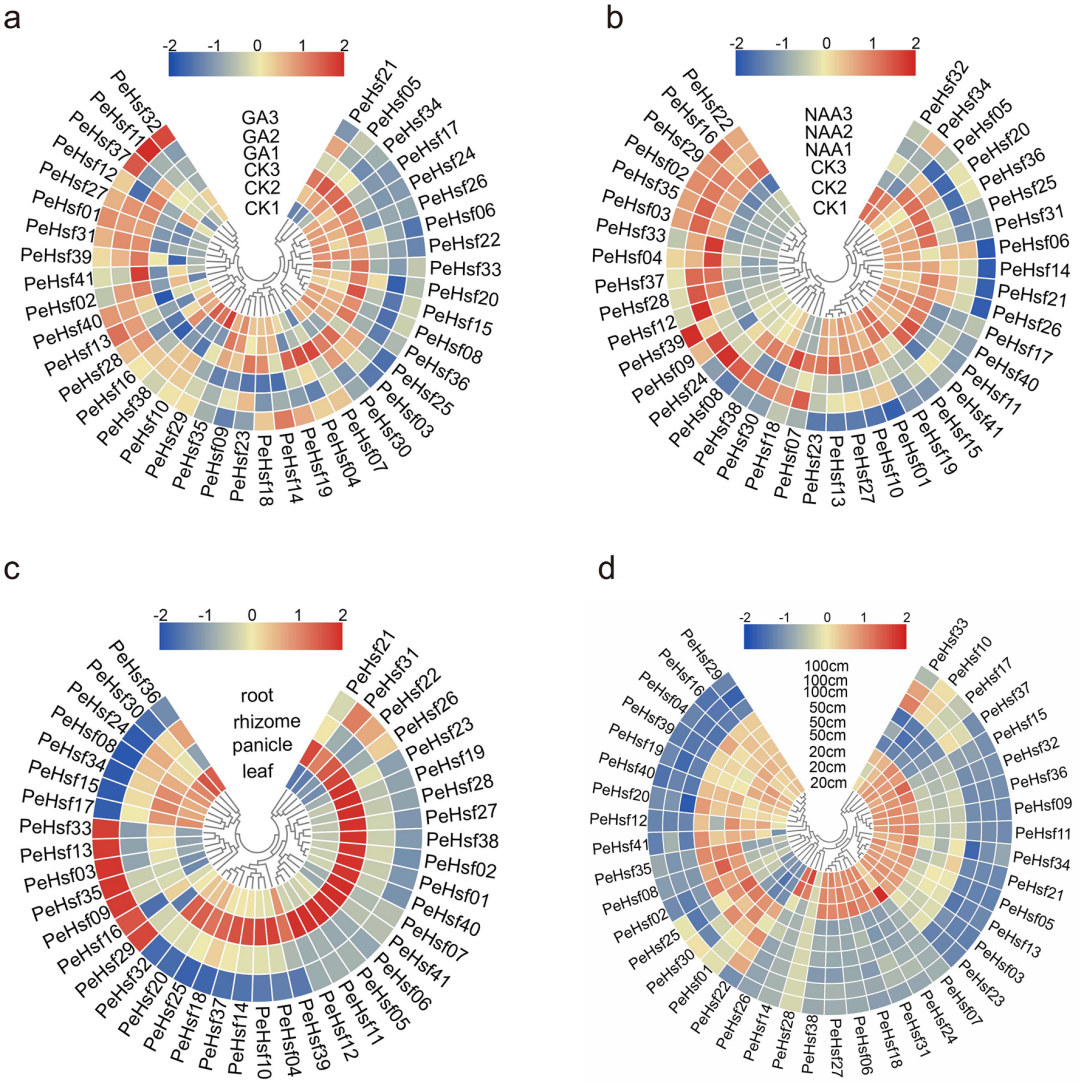
Heat maps of *PeHsf* gene expression (log_10_-transformed TPM values) in multiple tissues and in response to hormone treatments. (**a**) GA treatment. (**b**) NAA treatment. (**c**) Roots, rhizomes, panicles, and leaves. (**d**) Young bamboo shoots of different heights. CK, control group. Each tissue or treatment was replicated three times and its gene expression levels averaged. The relative expression levels are depicted according to the color scale, where a change from blue to red indicates transcript abundance from low to high. Amazing Heatmap in TBtools (v1.0697) is used to generate expression heatmaps.

We used published transcriptome data to investigate the expression patterns of the *PeHsf* genes (1) in seedling roots treated with gibberellic acid (GA) or naphthalene acetic acid (NAA), (2) in roots, rhizomes, panicles, and leaves, and (3) in young shoots of different heights (20, 50, and 100 cm). In (Fig. 10 and Supplementary Table S4), blue indicates low transcript abundance and red indicates high transcript abundance. Under GA treatment (Fig. 10a), the expression of *PeHsf17*, *PeHsf24*, *PeHsf26*, *PeHsf06*, *PeHsf22*, *PeHsf33*, *PeHsf20*, *PeHsf15*, *PeHsf08*, and *PeHsf36* was downregulated compared with the control, indicating that GA repressed the expression of these genes. By contrast, the expression of *PeHsf27*, *PeHsf01*, and *PeHsf31* was elevated under GA treatment but reduced under NAA treatment (Fig. 10b), indicating that the root expression of individual *PeHsf* genes differed between the hormone treatments. Similarly, *PeHsf* expression differed markedly among plant organs (Fig. 10c). In general, more than half of the genes were highly expressed in panicles, and four genes (*PeHsf27*, *PeHsf11*, *PeHsf10*, and *PeHsf41*) had expression levels greater than 100 TPM in this organ. Seven genes (*PeHsf33*, *PeHsf13*, *PeHsf03*, *PeHsf35*, *PeHsf09*, *PeHsf16*, and *PeHsf29*) were highly expressed only in roots, suggesting that they may participate in the development and/or function of bamboo roots. *PeHsf36* and *PeHsf30* were highly expressed only in leaves, and a large proportion of *PeHsf* genes were either not expressed or expressed only at low levels in the rhizome. Expression analysis of bamboo shoots at different germination stages (Fig. 10d) showed that many *PeHsf*s were expressed highly in 20-cm shoots, at intermediate levels in 50-cm shoots, and at the lowest level in 100-cm shoots. In general, these *PeHsfs* were specifically expressed during rapid shoot growth, suggesting that they may have important functions during this time.

### GO enrichment analysis of the *PeHsf* proteins

**Figure 11:**
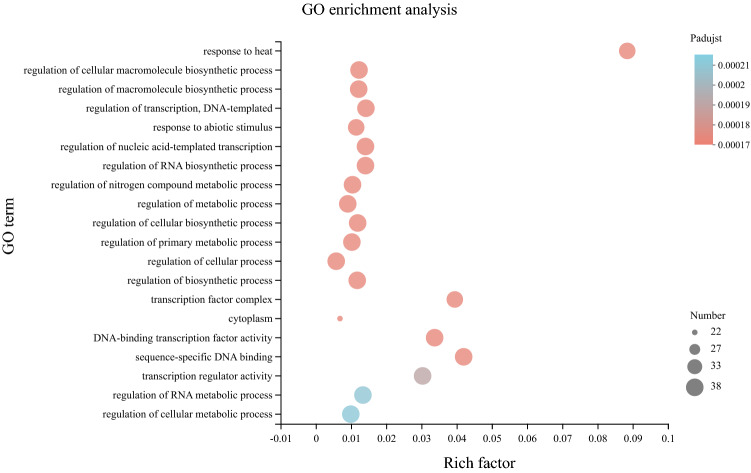
GO enrichment analysis of the PeHsf proteins relative to the GO database. The horizontal axis indicates the enrichment factor, and the size of the circle indicates the number of genes annotated with a given GO term. GOATOOLS (http://github.com/tanghaibao/GOatools) was used to assign GO annotations to HSFs, and Fisher's exact test was used to identify biological functions enriched in the PeHsfs relative to the full GO database. Visualization were performed using the Majorbio online platform (https://cloud.majorbio.com).

Plants have evolved complex mechanisms to perceive and respond to biotic and abiotic stresses, and HSFs are important components of these defense systems^2^. To further investigate the biological functions of the moso bamboo HSFs, we performed gene ontology (GO) annotation and enrichment analysis of the 41 *PeHsf* proteins (Fig. 11 and Supplementary Table S5). Eight molecular function, one cellular component, and eleven biological process GO terms were enriched in the PeHsfs relative to the complete GO database. Thirty-six proteins were annotated with the enriched GO term heat response, which had an enrichment factor of 0.09, confirming that heat response is a primary function of the PeHsf proteins. GO enrichment results suggested that *PeHsf* transcription factors were also involved in macromolecule biosynthesis, RNA biosynthesis, nitrogen compound metabolism, cellular biosynthetic processes, primary metabolic processes, and the regulation of RNA metabolic processes. The largest number of genes (37) were associated with the term “regulation of cellular macromolecule biosynthetic process”.

### Construction of a *PeHsf* protein interaction network

**Figure 12:**
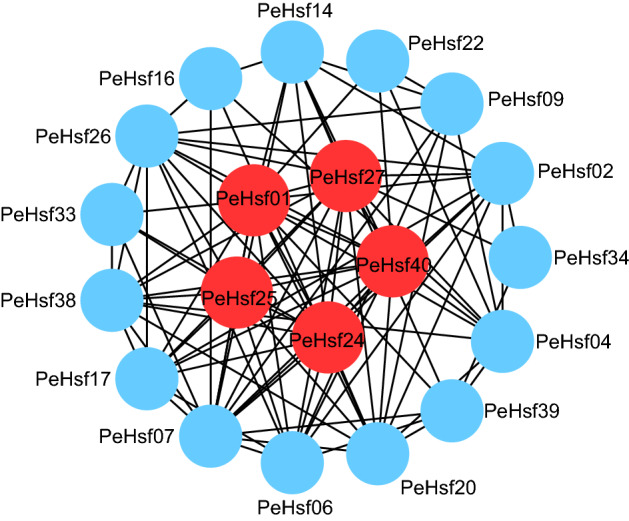
Interaction network of the HSF proteins in moso bamboo. Nodes represent proteins, central nodes are indicated in red, and black lines indicate interactions between nodes. Cytoscape (V3.7.1) (https://cytoscape.org/) was used to visualize the resulting network.

We used the STRING database to predict potential interactions among the PeHsf proteins (Fig. 12 and Supplementary Table S6). There were 19 nodes in the PeHsf protein interaction network, each of which interacted with multiple other nodes. Some proteins exhibited direct interactions, e.g. *PeHsf14* and *PeHsf16*, and others showed more complex multi-gene interactions, e.g. *PeHsf01*, *PeHsf16*, and *PeHsf23*. *PeHsf01*, *PeHsf24*, *PeHsf25*, *PeHsf27*, and *PeHsf40* were predicted to be central nodes, radiating numerous connections to other nodes.

### Homology modeling of *PeHsf* tertiary structures

**Figure 13:**
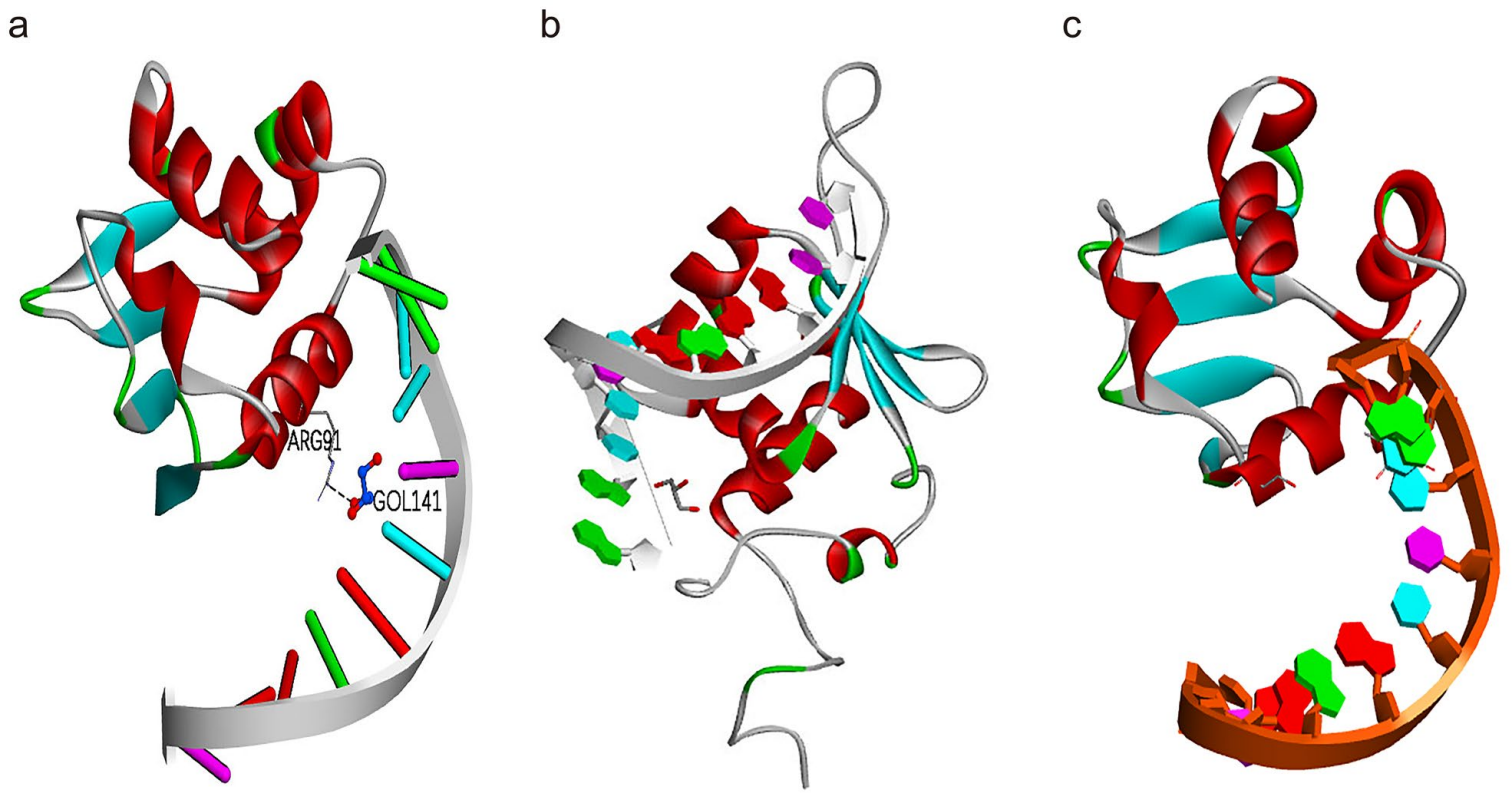
Predicted three-dimensional structures of the moso bamboo HSF protein sequences. (**a**) PeHsf15 was selected to represent the structure of the HsfA subfamily. (**b**) PeHsf28 was selected to represent the structure of the HsfB subfamily. (**c**) PeHsf26 was selected to represent the structure of the HsfC subfamily. Models were constructed using SWISS-MODEL. ARG, arginine. GOL, substrate. Predicted model structures were visualized and manipulated with Discovery Studio, version 2016 (BIOVIA) (http://www.discoverystudio.net/).

*PeHsf15* from subfamily HsfA, *PeHsf28* from subfamily HsfB, and *PeHsf26* from subfamily HsfC were selected for tertiary structural homology modeling. The three-dimensional structures of the three subfamilies shared a number of similarities (Fig. 13). The highly conserved DNA binding domain (DBD), a distinctive feature of HSFs, was visible as a spherical region formed from three α-helix bundles and four strands of reverse-parallel β-folded layers. Different sequences had different convoluted structures. In *PeHsf15*, ARG91 was linked to the substrate via hydrogen bonds (Fig. 13a). By contrast, *PeHsf28* (Fig. 13b) and *PeHsf26* (Fig. 13c), representing the HsfB and HsfC families, showed no hydrogen bonding with the substrate owing to their lack of AHA motifs.

## Discussion

HSFs are a specific class of transcription factors that play an important role in plant resistance to various stress injuries^58–60^. The *HSF* gene family has been studied in Arabidopsis^10^, tomato^15^, cabbage^16^, pepper^17^, poplar^18^, maize^19^, and rice^20^, and specific molecular functions of the HSFs have been validated in the model plants Arabidopsis and rice. However, *HSFs* have not previously been investigated in the economically important and widely distributed graminaceous species moso bamboo. At present, the genome draft of moso bamboo is almost complete, enabling the comprehensive characterization of important gene families^33,34^. Here, we identified 41 *PeHsf* genes from moso bamboo and classified them into three subfamilies (A–C) based on phylogenetic analysis (Fig. 1). Gene structure analysis revealed that some members within the same subfamily had structural differences. For example, *PeHsf31* in the *HsfC* subfamily contained three introns, whereas the other *HsfC*s had only one. *PeHsf31* may therefore have experienced the splicing or insertion of gene fragments during its evolution^61,62^. Nonetheless, similar conserved sequences and gene structures among *HSF* subfamily members suggests that genes within a subfamily may generally have similar biological functions.

The highly conserved plant HSF DBD is located at the N terminus, where it enables the precise localization and recognition of the heat stress element (HSE) in target gene promoters^63,64^. Here, multiple sequence alignment and secondary structure prediction showed that a DBD consisting of three α-helices and four β-folds was present in all PeHsf proteins (Fig. 2). Interestingly, other conserved domains were also present in some PeHsf proteins, and further experimental verification is needed to determine whether this is a manifestation of gene family functional diversification. Tertiary structure analysis demonstrated that the portion of the transcription factors that interacts with nucleic acids was conserved among subfamilies. HsfB and HsfC members did not contain AHA motifs and therefore probably lack transcriptional activation function, consistent with the results of previous studies^14^.

Gene duplication plays an important role in evolution by facilitating the generation of new genes and gene functions. There are three main evolutionary modes of gene duplication^10^: segmental duplication, tandem duplication, and translocation events. Segmental and tandem duplication most commonly underlie the expansion of plant gene families^57,65^. The number of *HSF* genes was significantly higher in moso bamboo (41) than in the model plants Arabidopsis (21) and rice (25), suggesting that gene duplication had occurred, consistent with previous reports of whole-genome duplication in moso bamboo^32,66^. We therefore performed intra- and inter-genomic collinearity analyses of the *HSFs*. Evolutionary analysis has shown that whole genome duplication (WGD) and segmental duplication play an important role in the expansion of the HSF gene family. Within the moso bamboo genome, there were 29 duplicated gene pairs among the *HSF* genes, including 27 segmental duplicate pairs and two tandem duplicate pairs. Segmental duplications therefore dominated the expansion of the *HSF* gene family in moso bamboo. By comparison, a previous study reported nine segmental duplicate pairs in 25 rice *HSF* genes^54^. Synteny analysis of the moso bamboo genome with four other sequenced plant genomes showed that there was significant collinearity of *HSF* family members between bamboo and the monocots maize and rice. Only a few *HSF* members were collinear between bamboo and the dicots Arabidopsis and pepper. This result is consistent with the evolutionary relationship between dicot and monocot plants.

There are four main fates of duplicated genes^67^. First, both genes may retain their original function, leading to functional redundancy. Alternatively, duplicates with the same ancestral function may gradually develop different functions in a process referred to as subfunctionalization. In a less common scenario, one of the duplicates may acquire a wholly new function (neofunctionalization). Finally, the intermediate evolutionary stage between subfunctionalization and neofunctionalization preserves genes that are essential for plant growth. We found that many *PeHsf* homologous gene pairs were expressed at similar levels (Fig. 10), suggesting that duplication of the *HSF* genes in moso bamboo has primarily resulted in functional redundancy.

Ka and Ks values were calculated for thirteen homologous, segmentally duplicated gene pairs. Ks values indicated that large-scale *PeHsf* gene duplication events had occurred between ~ 7.86 and 83.33 MYA. Interestingly, about 62% of the homologous gene pairs appeared to have undergone duplication far earlier than 12 MYA, consistent with the occurrence of a separate whole-genome duplication (WGD) event prior to the WGD previously reported to have occurred 7–12 MYA^32^. In addition, all the homologous gene pairs had Ka/Ks values less than 1, indicating that the *PeHsf* genes were subject to purifying selection during their evolutionary history.

Flowering is the most important life history trait in angiosperm plants, and bamboo is no exception. Unlike many other bamboos, moso bamboo produces a single flowering spike approximately every fifty years, blooms sporadically, and dies after flowering. Several studies have demonstrated that HSFs modulate the expression of stress-related proteins such as heat shock proteins (HSPs) that play important roles in plant stress response^68^. HSPs such as HSP70 and HSP90 are involved in flowering time regulation and vernalization pathways that promote flowering by inhibiting FLC expression^69^. Based on *HSF* expression profiling in different bamboo tissues (Fig. 10c), we found that many *PeHsf*s showed relatively high expression levels in the panicle. For example, orthologs of rice *OsHsfB2b* (*PeHsf27*, *PeHsf10*, and *PeHsf38*) were highly expressed in panicles, and a previous study has shown that OsHsfB2b acts as a negative regulator of plant drought response^20^. These *PeHsf* genes may therefore be involved not only in panicle development but also in the response of moso bamboo to drought stress. Some *PeHsf* genes showed little to no expression in any tissue tested, suggesting that they may act in other tissues or at other developmental stages. Interestingly, some *PeHsf*s were highly expressed in leaves but expressed at low levels in early 20-cm bamboo shoots, suggesting that they are mainly involved in cell differentiation and elongation, rather than in the process of lignification.

Multiple *cis*-acting elements located in gene promoters play a crucial role in signaling, and synergistic interactions among them can regulate complex biological processes. The drought-responsive MYB element was present in the promoter regions of almost all *PeHsf* genes, as were ABRE, MBS, P-box, TC-rich repeat, and SP1 elements, indicating that *PeHsf* expression is likely to be affected by abiotic factors such as high temperature, drought, high salinity, and light. Examination of expression profiles of seedling roots under NAA and GA treatments revealed that most *PeHsf* genes were expressed at different levels under treated and control conditions.

Some genes, (e.g., *PeHsf40*, *PeHsf31*, and *PeHsf13*) were upregulated by GA treatment, whereas others (e.g., *PeHsf33*, *PeHsf06*, *PeHsf03*, *PeHsf35*, and *PeHsf26*) were downregulated. Interestingly, the promoter analysis (Fig. 9) indicated that these genes all contained *cis*-elements involved in ABA response. In rice, multiple *HSF* genes are induced by exogenous gibberellin 3 (GA3) and abscisic acid (ABA)^70^, and multiple ABRE and P-box elements responsive to ABA and GA, respectively, were present in the *PeHsf* promoters. Promoter analysis and transcriptomic results therefore suggest that *HSF* genes are involved not only in the regulation of heat response, but also in responses to drought, salt, and various exogenous hormones (NAA, GA, and ABA), making them important transcription factors for plant resistance to multiple stresses.

## Conclusions

We systematically identified and analyzed 41 moso bamboo *HSF* genes and divided them into three subfamilies, each of which had similar gene structures and sequences. Evolutionary analysis indicated that segmental duplications associated with whole-genome duplication events were responsible for much of the expansion of the moso bamboo *HSF* gene family. Transcriptomic analyses confirmed that expression of some *PeHsf*s responded to exogenous GA and NAA application, and the high expression of other *PeHsf*s in 20-cm shoots suggested that they may function in the rapid growth and early development of bamboo shoots. The *PeHsf* gene family appears to be functionally diverse, with roles in bamboo growth and development, stress response, and the functions of specific tissues and organs. These results provide a foundation for the subsequent exploration of bamboo *HSF* gene functions.

## Supplementary Information

Supplementary Table S1.

Supplementary Table S2.

Supplementary Table S3.

Supplementary Table S4.

Supplementary Table S5.

Supplementary Table S6.
